# Herpes zoster as a diagnostic pitfall leading to an unwarranted cholecystectomy: a case report

**DOI:** 10.3389/fmed.2026.1854775

**Published:** 2026-05-29

**Authors:** Zi-Zhen Yin, Yu Liu, Shu-Zhen Pang

**Affiliations:** 1 Department of Hepatobiliary Surgery, Heze Medical College, Heze, Shandong, China; 2 Department of Internal Medicine, Central Hospital in Mudan District of Heze, Heze, Shandong, China; 3 Department of Internal Medicine, Heze Medical College, Heze, Shandong, China

**Keywords:** abdominal pain, acute cholecystitis, case report, cholecystectomy, herpes zoster, misdiagnosis, post-herpetic neuralgia

## Abstract

Some cases of herpes zoster infection present solely with abdominal pain in the early stage, potentially leading to misdiagnosis as cholecystitis and an unwarranted cholecystectomy. In August 2025, a 62-year-old female patient with a history of chronic cholecystitis presented with a 2-day history of right upper quadrant abdominal pain, without the characteristic vesicular rash. She was diagnosed with acute cholecystitis and underwent laparoscopic cholecystectomy. However, the subsequent development of clusters of vesicles on the right abdominal skin led to the diagnosis of herpes zoster. Herpes zoster infection can present with abdominal pain as its initial manifestation; this poses a formidable diagnostic challenge, potentially leading to misdiagnosis as acute cholecystitis and unwarranted cholecystectomy.

## Introduction

Herpes zoster is a distinct dermatologic entity with no established pathophysiological association with acute cholecystitis. However, some cases of herpes zoster initially present solely with abdominal pain in its early stage, which may mimic the presentation of acute cholecystitis, potentially leading to misdiagnosis and unwarranted cholecystectomy. In August 2025, a female patient with a history of chronic cholecystitis presented to our hospital due to abdominal pain in the right upper quadrant for 2 days. Based on clinical and imaging findings, she was initially diagnosed with acute cholecystitis and underwent laparoscopic cholecystectomy. However, within 24 h postoperatively, clusters of vesicles erupted on the patient’s abdominal skin, confirming the diagnosis of herpes zoster. Notably, the early radicular pain distribution overlapped precisely with the typical clinical localization of cholecystitis. This overlap led to initial diagnostic confusion, resulting in misdiagnosis and an unwarranted cholecystectomy. The gallbladder was mistakenly blamed for abdominal pain, highlighting the diagnostic pitfall when early-stage herpes zoster presents clinically as isolated abdominal pain.

## Case presentation

A 62-year-old woman presented to our hospital in August 2025 with a 2-day history of right upper quadrant abdominal pain. She denied associated fever, nausea, or vomiting. Her past medical history was notable for chronic cholecystitis (diagnosed > 15 years prior), which had not been formally managed. She had no history of jaundice, cardiovascular disease, hypertension, or diabetes mellitus. She reported no recent vaccinations and no family history of relevance to the current presentation. On physical examination, obvious tenderness was noted in the right upper quadrant, but no rebound tenderness was noted. At that time, the skin showed no erythema or vesicular lesions. A comprehensive set of laboratory examinations was conducted, the white blood cell count was 5.45 * 10^9^/L, C-reactive protein level 1.76 mg/L, potassium 3.35 mmol/L, and sodium 135.6 mmol/L, Liver function tests revealed alanine aminotransferase 15.6 U/L, aspartate aminotransferase 29.6 U/L, γ-glutamy transferase 6.4 U/L, alkaline phosphatase 69.0 U/L, total bilirubin 36.6 umol/L, and blood glucose 5.71 mmol/L. Other results related to thyroid function, kidney function, pancreatic function, electrolytes, cardiac enzymes, and cardiac troponin were within normal ranges.

**FIGURE 1:**
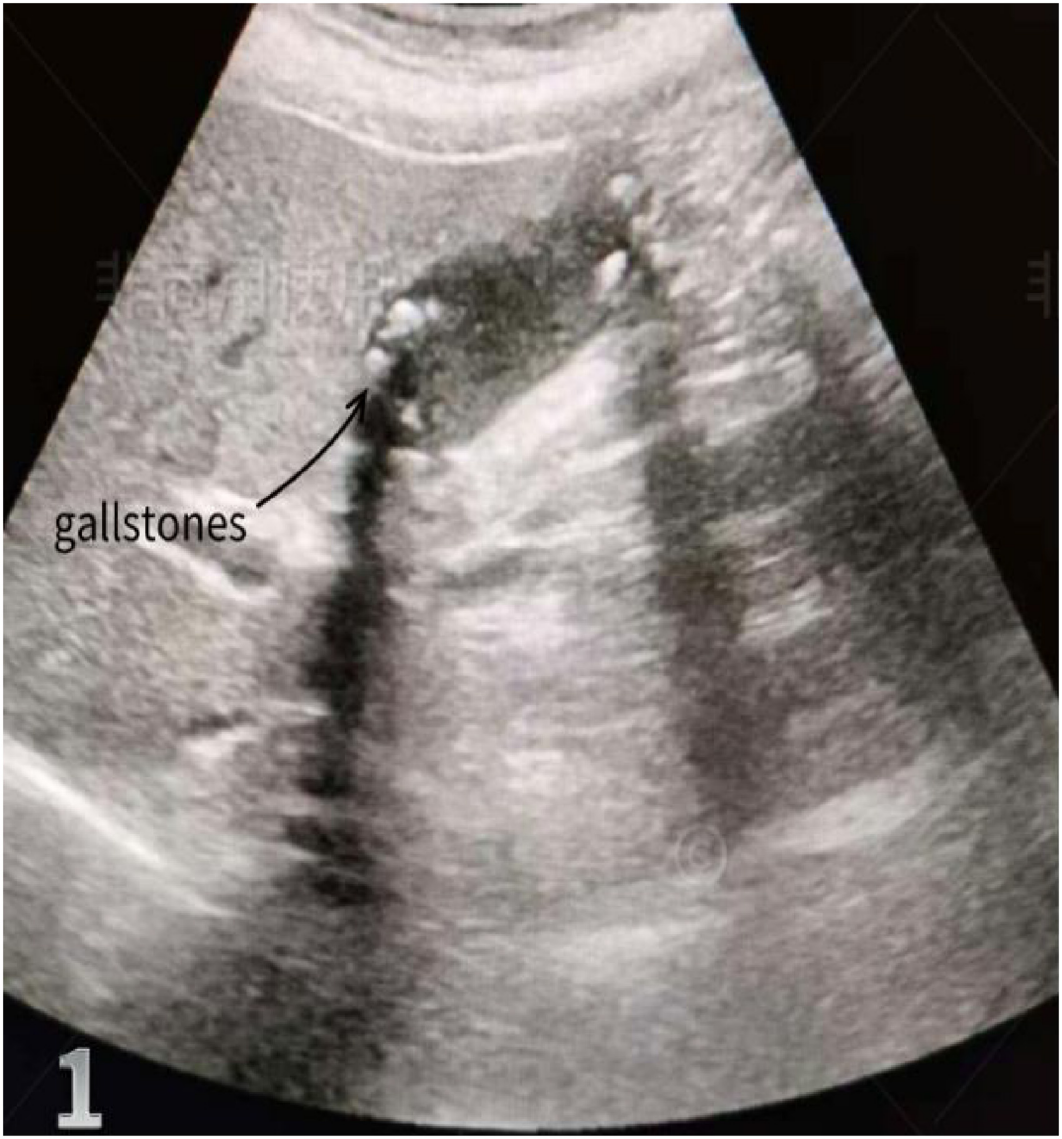
Ultrasound image showed gallstones.

**FIGURE 2:**
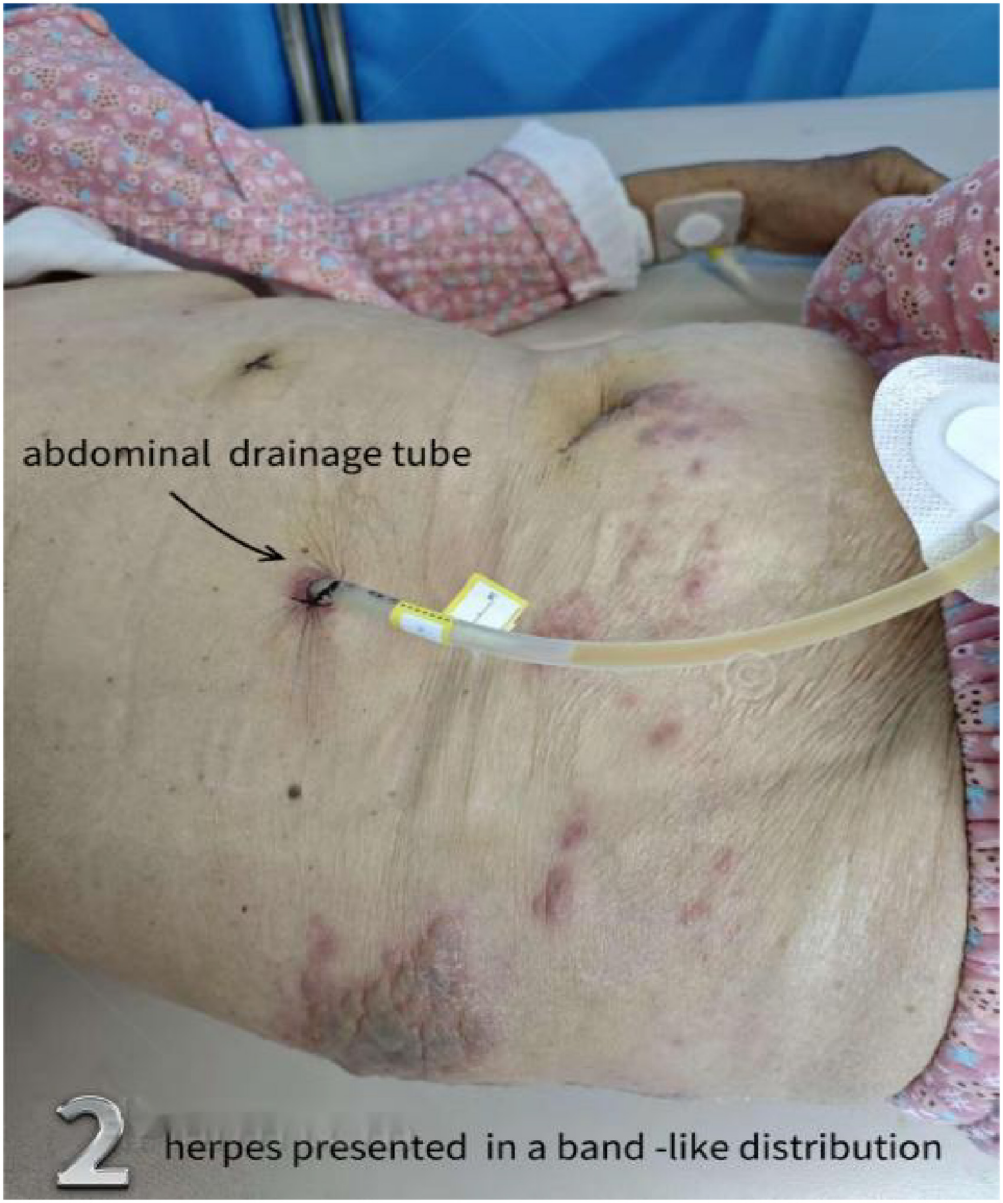
Redness and clusters of blisters on the abdominal skin.

Ultrasound images revealed gallstones (Figure 1). She was initially diagnosed with cholecystolithiasis and cholecystitis, which led to her admission. Based on clinical and imaging findings, the patient underwent laparoscopic cholecystectomy 1 day after admission. However, no typical inflammatory signs of acute cholecystitis were visualized intraoperatively; the gallbladder was removed due to the presence of gallstones. The following morning, the patient reported persistent pain in the right upper quadrant. We attributed the pain to incisional pain following the surgery. That afternoon, we were surprised to notice a distinct area of erythema on the patient’s right abdominal skin, accompanied by multiple clustered small blisters and pronounced localized pain. Shortly thereafter, the number of blisters on the patient’s abdominal skin continued to multiply (Figure 2).

A dermatology consultation was promptly arranged; based on a comprehensive clinical evaluation and characteristic cutaneous manifestations, herpes zoster was definitively confirmed. The patient initiated antiviral therapy and adjunctive analgesia. Within 2 weeks of treatment initiation, the vesicular lesions had markedly diminished, crusted over, and showed no new eruption; the patient was discharged upon completion of the full 14-day antiviral course. Although our misjudgment led to a cholecystectomy, our patient remained satisfied with the entire treatment process after the herpes zoster was controlled and raised no objections. Follow-up assessments were scheduled at 1, 3, and 6 months post-discharge. At the 1-month visit, the rash had completely resolved, leaving only post-herpetic neuralgia (PHN). By the 3-month assessment, residual PHN persisted, requiring continued medication for management. At the 6-month follow-up, PHN had resolved completely, with no recurrence of pain or rash.

## Discussion

In August 2025, a 62-year-old female patient with a history of chronic cholecystitis presented to our hospital with persistent abdominal pain in the right upper quadrant. The patient displayed no rash at that moment. She was diagnosed with acute cholecystitis and underwent laparoscopic cholecystectomy. However, postoperative pain persisted and a vesicular rash subsequently emerged, which was eventually confirmed as herpes zoster. The initial diagnosis was inaccurate, rendering the gallbladder a scapegoat. This case exemplifies the diagnostic challenge posed by herpes zoster and illuminates the clinical pitfall encountered when early-stage herpes zoster manifests as isolated abdominal pain. Herein, we present this case for further discussion.

Herpes zoster is an infection caused by the varicella-zoster virus, characterized by acute, unilateral, dermatomal cutaneous and neurologic inflammation (1). Clinically, it presents herpes and neuralgia in a unilateral bandlike distribution (2, 3). The rash is characterized by unilateral, grouped vesicles, distributed in a bandlike or arcuate pattern, usually not crossing the midline of the trunk. Affected regions include the thoracic, lumbar, cervical, and trigeminal dermatomes (4). Neuralgia is a core symptom, typically manifesting as stabbing, burning, or severe shooting pain 4–5 days before the presentation of skin lesions. The severity of pain does not necessarily correlate with the severity of the rash, and pain in the elderly may be more intense. Some patients may experience fever, headache, fatigue, swollen nodes, and other symptoms.

According to a systematic review of studies, the cumulative incidence has been estimated between 2.9–19.5 cases per 1000 population with female predominance (5). Diagnosis is primarily clinical and highly reliable when the characteristic rash is present. Rash typically resolves within 2 to 3 weeks. Herpes zoster-associated pain tends to resolve over time. PHN is the most common complication of herpes zoster (6), defined as persistent neuropathic pain that persists for at least 3 months after rash remission (7). Approximately 10–30% of patients will experience PHN (8), About 20% of patients experience neuralgia lasting longer than 6 months (9), which can lead to psychiatric disorders and depression, and can significantly impair quality of life (10). Acyclovir is the first-line agent. Oral antiviral therapy should be initiated within 72 h of symptom onset to suppress viral replication, alleviate pain, accelerate recovery, and prevent complications. Pain management should be individualized according to severity: mild-to-moderate pain may be addressed with standard analgesics, whereas severe pain often warrants adjunctive neuropathic agents such as gabapentin or pregabalin (11).

In this instance, the patient initially presented with abdominal pain in the right upper quadrant. At that time, the white blood cell count was not elevated, diverging from the typical laboratory profile associated with acute cholecystitis. We neglected to perform a thorough analysis of the clinical presentation, rushed into cholecystectomy based solely on ultrasound findings, and overlooked critical differential diagnoses. However, no typical inflammatory signs of acute cholecystitis were visualized intraoperatively, and abdominal pain persisted postoperatively. The subsequent emergence of distinct clusters of blisters led to a diagnosis of herpes zoster, ultimately confirming our patient had been misdiagnosed. Misdiagnosis stemmed from three interrelated factors: physicians’ cognitive biases favoring more common pathologies; insufficient clinical awareness of herpes zoster as a plausible differential diagnosis; and the nonspecific presentation of right upper quadrant pain, particularly when the characteristic rash is absent, delayed in onset, thereby complicating accurate site attribution.

Herpes zoster is usually accompanied by short-lasting stabbing pain in the involved sites (12). The pain in the waist and abdomen can radiate to the upper abdomen when intercostal nerves are involved, overlapping with the pain site associated with cholecystitis, and can be aggravated by palpation. Physicians are prone to cognitive biases when evaluating patients with right upper abdominal pain, often prioritizing common conditions such as cholecystitis and cholelithiasis, thereby neglecting rarer etiologies. For patients with a history of cholecystitis, physicians tended to ascribe pain to the gallbladder, neglecting the possibility of neuralgia. In severe neuralgia, it is easy to be misdiagnosed as biliary colic or radiation of cholecystitis before the appearance of a rash.

In the early stages of herpes zoster, patients may present with severe neuropathic pain alone, cutaneous findings may be absent, making differentiation from acute abdominal conditions exceptionally challenging. Some cases have been recorded in conjunction with herpes zoster infection initially presenting with abdominal pain (4, 13–18). This diagnostic dilemma is particularly pronounced in old patients, who face an elevated risk for herpes zoster and its complications (19), and are frequently misdiagnosed as biliary colic or cholecystitis, and sometimes lead to unwarranted surgical interventions.

To mitigate these cognitive biases, physicians should expand their diagnostic considerations when clinical findings appear contradictory. For patients exhibiting atypical symptoms without abnormal findings on routine examinations, rare conditions such as herpes zoster should be taken into account. In such scenarios, differential diagnosis must systematically consider the following conditions: acute cholecystitis, herpes zoster, pleurisy, intercostal neuralgia.

Cholecystectomy itself does not directly trigger herpes zoster. When elderly patients present with right upper quadrant pain, a comprehensive history must be obtained to determine whether the pain is electrical in nature or distributed along a nerve path. Careful inspection of the skin should be made, even if it appears normal. For tiny itchy or slightly thematous patches, which may be the early sign of herpes zoster. Early skin examination, ideally within 4 to 48 h of the pain onset, should focus on the development of erythema, papules, or vesicles, particularly in the thoracic lumbar region or along the intercostal nerves. Prompt referral to a dermatologist is advisable if the pain is inexplicable and of longer duration, to rule out herpes zoster.

Although the phrase “herpes zoster leading to cholecystectomy” sounds extremely rare, it is a real diagnostic pitfall in clinical practice. Physicians should maintain a high index of suspicion, particularly in elderly patients or those with a prior history of gallbladder disease. In such cases, the character and distribution of pain often hold greater diagnostic value than radiological findings. Physicians should systematically rule out neuropathic pain caused by herpes zoster when evaluating elderly patients presenting with right upper quadrant pain. Overlooking the diagnosis of herpes zoster can result in unwarranted surgical procedures and delay the initiation of vital antiviral therapy. This case serves as an important reminder for surgeons to be vigilant and consider differential diagnoses related to herpes zoster when imaging discrepancies arise. Surgical indications must be meticulously assessed. In cases presenting severe pain yet imaging reveals only cholelithiasis without inflammatory changes, physicians must proceed with caution and vigilantly consider the differential diagnosis with herpes zoster to prevent such errors.

## Conclusion

When herpes zoster clinically presents as isolated abdominal pain in the early stage, it is a diagnostic pitfall in clinical practice. The absence of visible skin lesions heightens the risk of misdiagnosis and may lead to unwarranted cholecystectomy.

## Data Availability

The raw data supporting the conclusions of this article will be made available by the authors, without undue reservation.
